# Young Creators: Perceptions of Creativity by Primary School Students in Malta

**DOI:** 10.3390/jintelligence11030053

**Published:** 2023-03-13

**Authors:** Margaret Mangion, Jasmin Antonia Riebel

**Affiliations:** Edward de Bono Institute for Creative Thinking and Innovation, University of Malta, MSD 2080 Msida, Malta

**Keywords:** creativity, self-perceived creativity, creative personal identity, education, primary school, creator, context, environment

## Abstract

Policymakers and employers insist that educational institutions prepare workforce-ready candidates fluent in the application of 21st Century Skills such as creativity. So far, only a few studies have explored the self-perceived creativity of students. This paper addresses this gap in literature by providing an understanding of how young students in upper primary feel about themselves as creative beings. Data for the present study was collected through an anonymous online survey that 561 students, aged 9–11 years and residing in Malta (EU), completed. In-depth responses were collected from a subset of the original sample made up of 101 students through an anonymous online form containing a set of questions. Data was analysed using regression analysis for the quantitative component and through thematic analysis for the qualitative part. Results indicate that, overall, students in Year 6 felt less creative than students in Year 5. Furthermore, findings show that the type of school attended impacted the students’ perception of creativity. From a qualitative perspective, findings led to insights into (i) the interpretation of the term creativity and (ii) the impact of the school environment and how timetabling impacted students’ creativity. The findings suggest that the student’s perceived creative personal identity and the concrete manifestations that they engage in are influenced by environmental factors.

## 1. Introduction

Creativity as an area of study has attracted increasing attention over the last few decades. Empirical research and theories have moved in a multitude of directions apart from the initial study of the person as a creative being. The value of creativity also found its way through practical paths of application through methods like creative problem-solving (Isaksen et al. 2011). Creativity and the desire to optimise it have come to the fore in occupational scenarios too (Kennedy and Sundberg 2020; Partnership for 21st Century Learning 2015) since it became a required characteristic to have on the job and also on a personal level (Beers 2016). With creativity featuring in the top places on the list, the 21st century skills have been identified with the intention to equip students and therefore future citizens of the world with a set of transferable skills that could be useful to address changing situations (Chu et al. 2017; Partnership for 21st Century Learning 2015; Voogt and Roblin 2012). What are referred to as ‘applied skills’ have been classified as very important for success at work (Casner-Lotto and Barrington 2006). Against this backdrop, the role of creativity and its development becomes imperative. This justifies the call by the late Anna Craft (2003) who urged scholars to consider creativity as an important part of education, a notion that is also supported by Gormley (2020). Linking creativity to education, Karwowski (2015) claims that the former “is among the most valuable human qualities achieved during the process of learning and teaching” (p. 165).

The aim of this study is threefold. Firstly, since there is an expectation that the future workforce becomes more creative and demands are made on students to enhance this skill, we thought that it is important to ask the students themselves what they think that creativity is. Secondly, it would be interesting to see if the students identify themselves with characteristics that are present in creative persons. Finally, how the environment impacts individuals in their engagement with creative practices is also of interest. Limited research has been conducted on implicit theories leading to how young students themselves interpret the term ‘creativity’ and their creative personal identity. This study hopes to build on the existing body of literature about implicit theories of creativity by exploring these research gaps also identified by Karwowski (2009).

## 2. Theoretical Background

### 2.1. Defining Creativity

Defining creativity has been an elusive task for many authors for decades (Plucker et al. 2004; Runco and Jaeger 2012). Although variations exist, there seems to be some consensus towards definitions with the inclusion of ‘novelty’, ‘originality’, ‘usefulness’ and ‘appropriateness’ (Amabile 1996; Runco and Jaeger 2012). Moving in a more action-oriented direction, Karwowski (2015) refers to creativity as a specific interplay between imagination, improvisation, and innovation. Corazza (2016) wrote about a concern with some definitions of creativity, primarily with Runco and Jaeger (2012) claiming that perceptions may be at play and may influence how an artifact is interpreted in terms of its originality and effectiveness. Mindful of the context within which this study took place, the definition of creativity offered by Plucker et al. (2004) is considered to be highly relevant, since they address the individual, the creative action and the context within which the creative activity takes place defining it as “the interaction among aptitude, process, and the environment by which an individual or group produces a perceptible product that is both novel and useful within a social context” (p. 90). This definition places a focus on the individual and his surroundings allowing a dynamic shift to the relationship between the creator, creation, and the context (Lubart 2017) within which such activity takes place.

Most of these explicit definitions emerged from studies following the work of academics and experts in the field mostly concerning adult participants (Plucker et al. 2004; Runco and Acar 2012). Runco and Bahleda (1986) claim that explicit theories are predominantly psychometric in genre however, as Lim and Plucker (2001) state, they do not explain how individuals perceive creativity while they are engaging in everyday activities. On the other hand, implicit theories of creativity are constructed by untrained individuals from their experiences. Their understanding of creativity is emergent as a result of their perception of the term (Niu and Sternberg 2002). To this end, implicit theories could also be perceived as subjective in nature. However, Gralewski and Karwowski (2018) state that implicit theories of creativity could be used to define traits linked to creativity including the judging of behaviour, while from a conceptual point of view Runco and Bahleda (1986) state that implicit theories are more easily tested in general. Following the aims of this paper, our objective is to formulate an understanding of what creativity means for the targeted sample population. Understanding differences that lie between explicit and implicit definitions of creativity may arguably provide grounds to enhance interventions that may facilitate human agency leading to creative behaviour. This may lead to an increased awareness of one’s creative personal identity.

In the literature creativity has been categorised in different ways. Rhodes (1961) categorised existing definitions at the time and coined the 4Ps framework. He included the *person* referring to individual characteristics related to creativity, product constituting of features that are appropriate and useful while being novel, process referring to cognitive activities in the creative endeavours and press referring to environmental elements that may lead to creative behaviour. Expanding on the 4Ps, the 7Cs of creativity were identified by Lubart (2017). Lubart captures a comprehensive illustration of the different dimensions through which creativity may be observed. Briefly, the 7Cs refer to Creators, referring to those who are engaged in the creative activity. Creating, that reflects the process related to the creative activity. Collaboration refers to third parties that may be involved in the creative activity thus adding an interpersonal dimension. Contexts refer to the physical environment where the activity takes place, while creations entail the product resulting from the creative activity. Consumption refers to the adoption of the creative product, and finally curricula is concerned with the education and development of creativity. This paper will make explicit reference to ‘creators’ and ‘context’ although it is not excluded that other facets of creativity might find their way in the discussion that ensues. It might be useful to observe that the 4C model of creativity (Kaufman and Beghetto 2009) could arguably be employed to explore the level at which the 7Cs might occur. Ranging from eminent creativity (Big-C) which is not a common occurrence in the population, and Pro-C which relates to more professional approaches to little-c creativity which involves everyday, practical creativity, and mini-c which is manifested through a sense of analysis and problem-solving this model categorises different levels of creativity effort. Little-c creativity is similar to everyday creativity in that it relates to activities that address issues such as mixing ingredients into a recipe (Richards 2010) that is different from what was previously available. In the case of mini-c creativity, Beghetto and Kaufman (2007) refer to an interpretive and transformative that links the process of leanring and creativity. By interpreting and manipulating information received new transformations manifested as creative ideas may emerge. Since the creative self-concept, which includes creative personal identity, refers to one’s understanding of how able they are to think and act creatively (Karwowski and Beghetto 2019), one may suppose that the rate at which creators engage with the contexts they find themselves in may impinge on the quantity and quality of activities related to little-c/everyday creativity and mini-c creativity. Supporting this argument is a recent study by Li et al. (2022). The authors investigated the link between the creative self-concept, and creative behaviour amongst others and found a positive relationship between the two.

### 2.2. Children’s Psychological Development and Their Understanding of Creativity

Over the last few years, the creative self has acquired considerable importance especially through the work of Karwowski (Karwowski et al. 2013; Karwowski 2015). Flammer (1995) states that self-beliefs are shaped over time as one goes through different phases. Following the argument by Flammer (1995), one may assume that the creative personal identity of young individuals emerges as they acquire life experiences. In Eysenck (2022) Piaget’s theory of cognitive development is outlined referring to schemata that are linked to different developmental stages of the child. Piaget claimed that between 7 and 11 years of age children typically reach the concrete developmental stage whereby they can start to solve problems in a logical way. The development of abstract thought follows this phase. At the age around 8 children also begin to innovate instead of imitating (Stricker and Sobel 2020). By contrast from a sociocultural perspective, Vygotsky (1962) viewed social interaction as the primary source of cognition and behaviour. Such interactions facilitate continuous processes rather than stages. The social and cultural aspects are given considerable importance in the shaping of personality through scaffolding. According to Kohlberg’s (1984) theory of moral development, by age 9 children start to acquire conventional morality where acceptance of social rules about what is right and what is wrong is predominant.

Some studies have explored how young students interpret creativity (Eshun et al. 2013; Karwowski 2015). Others have explored the self-perceptions of creativity by students (Deveci 2021; Falconer et al. 2018; Stricker and Sobel 2020). While Kupers et al. (2019) claim that understanding children’s creativity is very important for teachers, it may be argued that before we can start to understand children’s creativity, the children themselves need to know what it is and how they can nurture it. This may facilitate the cultivation of creative behaviour of young students.

Stricker and Sobel (2020) conducted three studies to understand how children aged 5–10 reflect on and understand their creativity as well as others’ creative actions, and the role that novelty and utility play. The first study consisted of semi-structured interviews with open-ended questions asking the participants what it means for them to be creative. Interestingly, the children’s examples of their own creativity showed to be more specific and relevant than those of others. In the next experiment, children and adults were asked to compare two different actions in terms of creativity and to justify their answers. According to this second study, the age around 7–8 years seems to be a transition point where the children’s perception of creativity and their appreciation of the novelty aspect did not differ significantly from adults anymore. The third study focused on utility instead of novelty as the second cornerstone of creativity, according to the standard definition of creativity (Runco and Jaeger 2012), with the result that here, older children underrated utility and perceived actions which were novel but not useful as more creative.

A further study exploring creativity in primary school children was conducted by Falconer et al. (2018). This research investigated the infamous ‘fourth grade slump’ which represents a drop in scores on creativity tests using four measuring scales. Although these studies are useful to understand a general picture when it comes to ‘quantifying’ creativity, they miss out on the possibility of delving deeper into the ‘how’ and ‘why’ elements of empirical findings. This further justifies the need for the current study since these qualitative aspects are addressed together with a more generic interpretation through quantitative means.

### 2.3. Creative Personal Identity

The self-concept is made up of the belief that a person may have about oneself. Rogers (1959) was one of the first psychologists to explore the self-concept and attributed three different components to it; the self-image, self-esteem, and the ideal self. More recently in her work related to everyday creativity, Richards (2010) addressed issues related to the development of the self-concept. In modern creativity literature, the role of creative self-beliefs, which represent a group of beliefs that make up the creative self of individuals, has come to the fore (Karwowski 2016) in relation to the important role it could play in the manifestation of everyday creativity (Richards 2010) and creative potential (Runco and Acar 2012). In relation to the creative self-concept, typically we find reference to creative self-efficacy (CSE) (Beghetto 2006; Karwowski et al. 2013; Tierney and Farmer 2002), creative-personal identity (CPI) (Karwowski et al. 2013; Jaussi et al. 2007), creative metacognition (Kaufman and Beghetto 2013) and self-rated creativity (Furnham et al. 2008).

In his work Erik Erikson (1968) established his theory of identity. According to the psychologist, identity is shaped throughout life and is categorised into eight psychosocial stages. Essentially, identity provides the individual with perceptions in relation to the self and the interaction with others. In more recent studies Randel and Jaussi (2003) differentiated between social identity and personal identity with the latter showing an increased stability overall (Brewer and Gardner 1996). The CPI of individuals entails the attributed importance of the construct in relation to the person’s identity (Jaussi et al. 2007). In a study conducted by Karwowski and colleagues in 2013, CPI was found to have a strong positive relation with Openness to Experience as a psychological construct. In keeping with the focus of this study, since Karwowski and Barbot (2016) highlighted that the creative self-concept is relatively well developed by the age of 10 and since Brewer and Gardner (1996) referred to the stability of personal identity, the pertinence in exploring how young students feel about their CPI seems to gain traction. Previous studies have focused on adult populations involving university students (Kaufman and Beghetto 2009) and working individuals (Tierney and Farmer 2002). It is only more recently that studies started to shift the attention to younger populations (Karwowski 2015). This allows for an enhanced scope for the current paper.

### 2.4. Schools and Creativity

There have been contradictory accounts of how school and the school environment impact student creativity. The TED talk by the late Sir Ken Robinson about how schools kill creativity (Robinson 2006) and his writings attracted considerable attention over the years. Focusing on the creativity gap, Runco et al. (2017) studied a population of undergraduate students and found that they were less creative at school than when in other contexts. They found that creative potential was evident outside of school but was not used once on the school premises. This could be possibly linked to the lack of autonomy offered in school settings. Their sample also indicated that students were more creative when they were alone rather than being with other students. In the meantime, studies and scales have been put forward to establish the creative climate in schools (Richardson and Mishra 2018). Studies like the ones highlighting the fourth grade slump (Kim 2011) still attract considerable attention although more care should be taken about the contextual elements that may lead to the significance of statistical results. Torrance (1968), noticed the fourth grade slump when exploring fluency, flexibility, originality and elaboration in a sample of third and fourth graders over a number of years. Kim (2011) found that the production of ideas increased until the third grade but remained constant between the fourth and the fifth grade followed by a decrease. Kim associates this decrease with the possibility that young students might self-impose judgment and fear inaccuracies or potential issues with the appropriateness of their responses. Karwowski (2022) reviewed literature outside the field of creativity in search for factual references about how school may kill creativity. His search revealed that this claim does not depict the true nature of the phenomenon under discussion.

Davies et al. (2013) reviewed 210 documents commissioned by the Learning and Teaching Scotland. Their search led to the emergence of a number of factors that concern the context the students were receiving their education in thus adding relevance to the environment as an important factor to take note of when promoting creativity. Exploring the environment from different perspectives, namely physical, pedagogical and external involvement, among these factors one finds reference to the flexible use of space and time, availability of resources, working in different environments outside the formal classroom, and the inclusion of elements of playfulness and gamified activities. Moreover, the review found that soft skills like the formation of respectful relationships between the young students, their teachers and opportunities for group work, and the involvement of external entities were also beneficial to the development of skills related to creativity. The role of the teachers is highly important when establishing environments that are conducive to the creativity of young students while at school Al-Dababneh et al. (2019). Gormley (2020) explored creative environments in relation to risk-taking, autonomy, novelty and flexibility from the perspective of teachers. He found that creative environments are sometimes influenced by examinations and expectations. He goes on to note that these findings are significant in view of the emphasis currently placed on education and the need to foster creativity. Apart from these conclusions, students’ perception of how their teachers relate to them greatly influences how ththeir classroom experience unfolds. As a consequence, teachers can make a difference by supporting their students’ creative expression through practices and procedures in the classroom that encourage taking intellectual risks such as expressing novel ideas (Beghetto 2006). Exploring the primary physical education perspective on education, Konstantinidou et al. (2014) found that only 36.5% of their sample agreed that primary school students are offered a lot of opportunities to manifest their creativity in school. The authors claim that sports including basketball, volleyball, gymnastics and sports skill practice offer the possibility for creative activity.

Of particular interest is how these influences may be catalysed by the school environment in relation to theoretical concepts of creativity. Taking into consideration the 4C Model by Kaufman and Beghetto (2009), it is pertinent to consider how the environment may play a role in the development of the different ‘Cs’ in the model. It is assumed that one can build on the engagement and proficiency experienced through different ‘Cs’ starting with the development of mini-c creativity all the way up to Pro-C Creativity (Karwowski et al. 2022). Abilities to solve problems and the combination of different types of information could arguably be addressed as early as pre-school age and developed throughout the years of formal schooling. The facilitation of the above-mentioned aspects related to the environment may provide fertile grounds where mini-c and little-c could be developed and nurtured during the primary years through creative learning (Beghetto 2016, 2019) with mini-c creativity being critical to creative learning due to its potential to develop ideation and problem-solving skills (Karwowski et al. 2022). The authors go on to claim that “making creativity more salient in the school environment not only requires enhancing students’ creative abilities but also building their creative selves.” (p. 244) Grohman (2019) conducted an experiment where she worked on cultivating little-c and mini-c creativity in students in higher education by teaching for creativity resulting in journals and a creative project. These hands-on tasks provided space where the students could practice skills related to problem solving and the application of knowledge as for little-c and mini-c creativity. This section offered an overview of some of the research that has been exploring different facets of creativity in schools over the years. The current study is interested in how young students perceive the school environment to facilitate or inhibit their level of creativity during the school day.

### 2.5. Research Questions and Hypothesis

Following the review presented above, one might expect that students may have an idea of what creativity may mean and for them to have a general conceptualisation of themselves and their creativity through their creative personal identity. The following research questions which will be explored through a qualitative study and hypothesis which will be tested on a larger sample are proposed:

RQ1: How do Maltese primary school students perceive creativity?
RQ2: How do students aged 9–11 perceive their creative personal identity?
RQ3: To what extent do students aged 9–11 acknowledge their creative personal identity?

**H1.**  *Students aged 9–11 have a positive perception of their own creative personal identity.*

## 3. Materials and Methods

### 3.1. Context and Samples

The present study took place in Malta, a small island state in the European Union. The total population on the island at the time of data collection was around 516,100 (NSO 2021). In Malta, school is compulsory between the ages of 5 and 16 and students may attend one of three types of schools, state schools run by the state, church schools run by the Secretariat for Catholic Education, and independent schools which are privately run. Primary school education covers Years 1 through to Year 6 where students sit for a national benchmarking assessment. Fifteen schools were invited to take part in the study with eight schools accepting the invitation. The selection criteria used during the recruitment of participants were related to the aim of the study, primarily for participating students to be between the ages of 9 and 11, which in general refers to children in their fifth and sixth year of primary education in the loval context respectively. Here grade five is equivalent to grade four in the American school system.

This research project is made up of two studies. For both studies, following adherence to all the necessary ethics procedures, clearance was obtained from the University of Malta’s Research Ethics Committee and the Directorate for research, lifelong learning and employability within the Ministry of Education, Secretariat for Catholic Education and Independent schools. The headmasters of the selected schools agreed to act as intermediaries in the dissemination of the information letters and consent forms. Signed consent was obtained from the parents or legal guardians and sent directly to the researcher. All data was collected online between April and June 2021 since access to schools was not allowed due to the mitigation measures that were in place to control the spread of the COVID-19 virus. Participants were presented with an online form that included three sections. Section one aimed to collect demographic data, section two was aimed to collect responses related to CPI adapted from Karwowski et al. (2018), and section three contained open and close-ended questions. A copy of the questionnaire can be found in the Supplementary Materials. The primary researcher accessed each class virtually and read out each question to all groups to avoid potential misinterpretations.

### 3.2. Study 1

For Study 1, the data obtained anonymously through submission via Google Forms was analysed using the Statistical Package for Social Science (SPSS), following a data cleaning process. 561 participants aged between 9 to 11 attending primary schools in Malta took part. The students attended either Year 5 (n = 298; 53%) or 6 (n = 263; 47%) of either a state school (n = 84; 15%), a church school (n = 260; 46.3%), or an independent school (n = 217; 38.7%). The schools of which two were state schools, three church schools and another two independent schools were selected through convenience sampling. This sample can be seen as representative for the Maltese population. The results, however, are not generalizable beyond primary aged Maltese school students. The Google Form comprised two main sections. In Section A respondents answered questions related to demographic data while in Section B the questions about CPI was presented. The questions for CPI are presented in Table 1.

The questions relating to the CPI in the Short Scale of Creative Self (SSCS) questionnaire by Karwowski et al. (2018) were used to collect data. The instrument has properties of high internal consistency as well as high reliability of measurement over time. Five items, including “I think I am a creative person” or “Creativity is an important part of myself” (Table 1), with a Likert-scale ranging from 1 (no way) to 5 (yes), were used to measure the students’ trait-like Creative Personal Identity. Students were also asked to give some demographic details and to answer additional questions such as what their favourite subject in class is.

### 3.3. Study 2

Study 2 involved 101 students, all of whom also participated in study 1, and who attended two church schools. The sample comprised of Year 5 (n = 48; 45.7%) or Year 6 (n = 57; 54.3%). In this study, female students were in the majority with 57 (56.4%) female participants and 44 (43.6%) male participants.

The researchers developed a set of questions (Table 2) related to creator and context as part of the 7Cs framework by Lubart (2017) to explore the students’ perceptions of creativity on its own as well as related to the school environment. We outlined five open-ended questions to give respondents the opportunity to express themselves and two close-ended questions. The latter included ‘are you creative?’ and ‘is the school a place where you can be creative?’ These questions were purposely asked since we were hoping to first obtain a yes/no answer followed by an elaboration in the question that followed.

### 3.4. Data Analyses

Data collected for Study 1 was analysed using the Statistical Package for Social Science (SPSS) using correlations and regression analysis. According to Miller and Acton (2009) a regression analysis is suitable and congruent with linear models. This appeared to be suitable in relation to the current study. Data collected from Study 2 was analysed through thematic analysis using the method by Braun and Clarke (2006). The authors define thematic analysis as “a method for identifying, analysing, and reporting patterns (themes) within data” (p. 6).

The thematic analysis for the present project is informed by an essentialist/realist world-view, and has been analyst-driven, following a theoretical approach as opposed to an inductive analysis, since the researchers were especially interested in two areas: *Creator* and *context*. This led to a number of themes explicitly related to these two areas, and enabled the researchers to gain insight into the predetermined research questions.

Following Braun and Clarke’s (2006) guide to performing thematic analysis in six phases (Table 3), a semantic approach was employed where the researchers stayed close to the explicit meaning of the data without “looking for anything beyond what a participant has said or what has been written” (Braun and Clarke 2006, p. 13).

Coding of the qualitative data was carried out using NVivo 12. With the two overarching themes ‘creator’ and ‘context’ already set, the researchers coded the whole data set twice: First, in relation to the participating students as creators, and a second time, with emphasis on the context in which creativity took place (or did not). In both instances, the process involved progressing from the descriptive level of the initially developed codes to summarizing the detected patterns into categories and, furthermore, interpretation of these patterns by defining and naming sub-themes. During this process, a short description for each code was developed (Table 4) before ideas to cluster the codes were brainstormed. The results were then transferred to a spreadsheet which was vital for the process of reviewing and refining the sub-themes. Phase four and five of Braun and Clarke’s (2006) approach happened collaboratively and interactively which gave the researchers the opportunity to bring together their unique understandings of the obtained results which will be described in the following section.

## 4. Results

### 4.1. Study 1

#### 4.1.1. Descriptive Statistics and Preliminary Findings

Over half the participants were male (n = 325; 57.9%), while almost half of the sample had one sibling (n = 273; 48.7%) while 16.6% of the participants had two siblings, and only 5.8% lived in families with three or more children. 16.9% said that they did not have any brothers or sisters. When being asked about their favourite subject, around 39% of the participants listed Mathematics as their first favourite subject. 15.9% of the students liked Physical Education (P.E.) the most, while 9.1% named Science and 9.4% English as their favourite lessons at school. Around 22% of the participants had either one or both of their parents working in Public Administration, Education or the Health Sector.

When looking at the students’ Creative Personal Identity, all items from the SSCS scored very high with the most dominant item being “Being a creative person is important to me” with a mean score of 4.47 out of 5. “Finding a solution is a characteristic which is important to me” had the weakest mean score amongst all five items but was still exceptionally high with a mean score of 4.26. When looking at the Creative Personal Identity scale as a whole, the average is 4.37 (Table 5).

#### 4.1.2. Data Analysis

A correlation analysis that was initially carried out may be found in the supplementary material accompanying this article. This analysis did not indicate any relation between the type of school, the mode of delivery during the school term, or the family situation (e.g., the number of siblings) and any item of the CPI. However, it seems more likely for only-children to attend an independent school (*p* < 0.05). Participants who named English as a favourite subject were likely to not name Mathematics as a favourite subject (*p* < 0.05). Another negative correlation was found between Science as a favourite subject and Mathematics (*p* < 0.05). Several correlations were found between the five items of the CPI scale of the SSCS (Table 6). These inter-item correlations are in line with a study by Karwowski et al. (2018) that confirmed high internal consistency of the SSCS after its development.

Subsequently, regression analysis was performed for different models. To see overall effects, a regression was run with all three types of school entered in the model as independent variables. This decision is based on findings by Briguglio et al. (2022) who looked into creativity in relation to different school types in the Maltese school context as well and found church schools to be negatively related to students’ creativity.

#### 4.1.3. Summary of the Findings

Through the analysis of our findings it emerged that Year 6 students feel less creative than Year 5 students (B = −0.159, *p* < 0.05). Moreover, state schools are significantly and positively associated with CPI (B = 0.223, *p* < 0.05), while no significant effect was found for church schools (Table 6). In this model, the Independent School variable was excluded by SPSS due to multicollinearity with the tolerance being 0. The students’ gender and number of siblings did not show any impact.

### 4.2. Study 2

The emerging findings were divided into two overarching themes; creators and context. Sub-themes have also emerged as indicated in the findings below (Table 7 and Table 8).

#### 4.2.1. Theme: Creators

The creators represented by students aged 9–11 emerged as a strong overarching theme in the study. An illustration of the subsequent sub-themes is displayed in Table 8. Overall, participants showed a very nuanced understanding of creativity even at their young age, ranging from providing implicit definitions of creativity to perceptions of creativity related to the academic definition of the term, covering aspects such as novelty, originality, and usefulness as illustrated in the extracts below:

*“When you think of an idea which is different from the ideas of others, ideas which are unique, and when you let your imagination run wild.”*

—Participant 2 (Female, Year 6)

*“Something that not everyone can think of”*

—Participant 107 (Female, Year 5)

*“The ability to think unique thoughts and to do what no one else has done”*

—Participant 15 (Female, Year 6)

*“To do something in your own special way”*

—Participant 125 (Female, Year 5)

As seen above (Participant 15), originality was also associated with ‘uniqueness’ in a number of cases and in one case ‘special’. The terms ‘imagination’ or ‘invention’ were also often mentioned when the students were asked to define creativity. Imagination and other cognitive functions including ‘thinking’, ‘use your brain’, ‘dreaming’, were attributed to creativity when participants defined creativity on a number of occasions. These comments were often linked to action-oriented tasks which involved thinking and doing such as building and creating objects. Although there appeared to be a predominant link between creativity and the arts and crafts, creating as a process within a constrained environment and involving problem-solving skills also emerged as a result of the process of imagination and creation when linked to creativity as indicated in the extracts below:

*“I made it using my imagination and from things I found on the ground”*

—Participant 142 (Female, Year 5)

*“I think it is a way to help people or fix situations”*

—Participant 22 (Female, Year 6)

*“I think creativity is when someone invents something from scratch”*

—Participant 639 (Male, Year 6)

Creativity was also referred to as “something important to have” and “useful in life”, “fun” and as a form of self-expression to externalise feelings and ideas and in order to develop and in relation to personal growth:

*“It helped me believe in myself more”*

—Participant 6117 (Male, Year 6)

The importance of creativity appears to be linked to the consequential psychological development of the students such as building of self-confidence for instance through the praise and acknowledgement the students received for being creative, for example, when their teachers, peers or families liked and approved of what the respondents created. Moreover from a more social perspective, some students remarked that creative ideas and actions can impact our surroundings though problem-solving:

*“leads the world to be a better place”*.

—Participant 119 (Female Year 5)

Besides exploring the meaning that young students give to creativity, it was also useful to find out when they were creative most and under which conditions. It was interesting to note that students seem to sometimes confuse creativity with creative writing (essay writing) which takes place in languages. This could be due to the fact that on day-to-day occasions, creativity or everyday creativity are not explicitly referred to other than during art lessons referring to artistic creativity and when creative writing tasks are set during language lessons. Moreover, most respondents associated creativity with activities which are extracurricular in nature most of the time.

*“When I do a new trick of football”*

—Participant (Jayden Vella (Male, Year 5)

“*During my free time*”

—Participant 636 (Male, Year 6)

*“I am creative most when I’m playing, drawing, doing crafts and when I’m day dreaming.”*

—Participant 104 (Female, Year 5)

*“When I am doing creative writings, during breaks when I play with my friends, when I do a craft and when I do the steam lesson work.”*

—Participant 636 (Male, Year 6)

Moreover, overlapping with the overarching theme represented by ‘context’, students seem to attribute conditions that facilitated creativity when asked ‘when are you creative most?’. It was evident that they placed considerable value on autonomy, support and feedback in the form of input and the concept of time namely in making the time to think and to play. A request for quiet spaces and silence also emerged strongly in the responses while an element of intrinsic motivation emerged through responses that explicated excitement and anticipation.

*“When I am in a valley and there would be silence”*

—Participant 6326 (Male, Year 6)

*“I am mostly creative when eager to do something I’m really excited for”*

—Participant 617 (Male, Year 6)

In relation to their own creations, when asked ‘what is the most creative thing you ever did at school?’ and ‘why do you think it was creative?’ examples of self-perceptions and manifestations of self-belief in the ability to be creative emerged as can be observed below:

*“Think and finish before the rest, coming up with new ideas. I think because not everyone can come up with new ideas”*

—Participant 535 (Male, Year 5)

*“My social studies project book in year 4. Mine was the only one like it”*

—Participant 5321 (Male, Year 5)

*“An object for the STEAM lesson where I had to create something without spending a lot of money. I made a Christmas tree out of old school plastic bottles, cloth and handmade decorations using reusable things. Because I built it all by myself without looking at any pictures or examples. I also helped in reducing waste”*

—Participant 636 (Male, Year 6)

*“When I read extracts from George’s Marvellous Medicine and Cinderella to the class during drama lesson. Because I used my own expressions and pacing and I felt proud of doing well”*

—Participant 15 (Female, Year 6)

*“I think the most creative thing I did at school was invent a one-word story game to pass the time while we waited for school to start. I think it was creative because no one thought of it an because everyone thought it was fun”*

—Participant 2 (Female, Year 6)

#### 4.2.2. Theme: Context

This overarching theme also emerged prominently in our findings. It was split into two sub-themes; ‘different places where the students are creative’, and ‘stimulation through the environment that fosters the student’s creativity’.

When asked ‘What would help you to be more creative while at school?’ the respones varied however there was a strong predominance for ‘more time’. More time was linked with thinking—“more time to think”, more time to interact with others, more time for craft, drama and music, and longer breaks. Some responses where more concrete in nature:

*“To discuss topics with friends”*

—Participant 133 (Female, Year 5)

*“When we’re in a lesson that we make things”*

—Participant 128 (Female, Year 5)

*“To be given more constructive free-time”*

—Participant 538 (Male, Year 5)

*“I think if there are more ‘games’ related to the subject. Ex. In social studies you have to act a scene”*

—Participant 6124 (Male, Year 6)

*“More science experiments”*

—Participant 102 (Female, Year 5)

It was noticeable how the element of noise appeared to be important for the participants. Responses included requests for more quiet spaces, “when the class is really quiet”. Other responeses included:

*“It will help me be more creative if there wouldn’t be as much noise.”*

—Participant 27 (Female, Year 6)

*“When around me is very quiet and calm”*

—Participant 97 (Female, Year 6)

The physical environment and its relationship with their creative activities, appear to bear considerable importance for the young respondents. The conditions appear to bring together a set of criteria that could make the environment more conducive to creative possibilities which often fall under the guise of social form of context. Clear distinctions were noticed in the responses registered when students referred to creativity while at school or at home. At home, the environment is less rigid and time is not controlled by the timetable or a bell. This appeared to allow the students more opportunities for engagement with creative activities. At school, specific subjects like Science and Physical Education were named often besides classes that are creative by nature, such as Creative Writing, Drama, Art or Music lessons. In general, the students wished for more creative activities during school (“*Creativity lessons would help me be more creative*”, Participant 6312, Year 6, male).

To conclude our analysis we found that the response below captures beautifully the student’s perception of creativity, his self-belief about creativity and an acknowledgement for the need of creativity in everyday life:

*“When you use your brain and come up with something creative*

*On a scale from 1–10, I give myself an 8.5 (I do think I am creative)*

*I am creative most when I have spaces to attack in the midfield in football*

*Yes—Without creativity you cannot do a lot of the subject”*

—Participant 6124 (Male, Year 6)

## 5. Discussion

Studies about the definition of creativity and CPI in adults are abundant in literature. In view of the importance attributed to creativity in the list of 21st Century Skills, it was thought that gaining a better understanding of how a future generation conceives the concept and how they feel about themselves as creative persons was a timely exercise to execute.

The aim of this study was to gain an understanding of what meaning young students attribute to creativity, to understand if they identify themselves as creative individuals and to explore the impact of the school environment on their creativity. A review of the literature led to the realisation that creativity is an important part of education (Craft 2003; Gormley 2020) and that it can be achieved during the learning process (Karwowski 2015). This further enhances the need to understand creativity as a construct from the point of view of young students as recipients and contributors to processes that may lead to creative outcomes.

In line with explicit definitions of creativity (Amabile 1996; Runco and Jaeger 2012), the respondents attributed originality and novelty to creativity. Moreover, there was an emphasis on the uniqueness of creative activities. From a more personal perspective it was interesting to observe more action-oriented definitions related to thinking and doing thus supporting the view expressed by Plucker et al. (2004). Thinking as a process also emerged noting the fact that the young participants acknowledge cognitive processes in some way. This is also indicative of their psychological development (Stricker and Sobel 2020). Focusing on the creators, namely young students in primary school and the context; of their respective schools, we tried to better understand how the participants identified with creativity in an effort to find elements that might be assumed or overlooked by adults who run the education facilities. Overall, the findings indicate that Year 6 students feel less creative than Year 5 students. A number of factors might account for this including the stress that the national benchmark examinations may impose on the students. Among the key findings we found that the students in general seem to believe that they are creative individuals and that creativity is important for them. Interestingly, many of the students who identified themselves as creative seem to have Mathematics as their favourite subject, but not science which at that age is still experiment based and very generic. Although the students in general felt that creativity is important for them, solution finding was not as strongly correlated.

Looking at Study 2 which involved students who also took part in Study 1, it was striking to see how clear the notion of time is to the respondents. Time was identified as one of the main sub-themes in relation to requests for more time to think and to engage with activities that promote the creative process including problem-solving. This is in contrast with findings from Konstantinidou et al. (2014). Echoing Davies et al. (2013), a review of pedagogical approaches that may enhance the school environment to make it more conducive to creative activity is highly relevant to promote little-c creativity (Kaufman and Beghetto 2009). The findings of the current research indicate that students seem not to have enough time for trial-and-error activities due to the highly structured curriculum. Therefore, in support of the claims by Craft (2003) and Gormley (2020), creativity may still find it difficult to penetrate the thick walls of the curriculum and rigid syllabi that might adopt traditional teaching.

The psychological development of the respondents had an important role in this study. As outlined by Karwowski (2015) the self-concept is fully visible by 10. It was insightful for us as reaserchers to observe the level of understanding and appreciation of the self-concept of the students. The findings in fact seem to indicate the need to invest further in self-perceptions through the provision of activities and reflective practices that the students can engage with. Carrying on with issues related to ‘time’ and moving away from academic performance and the influence that it may have on the self-perception of individuals, it was clear that the students do not have many opportunities to think about their own self-development.

When exploring the context, it was noticeable, how action-oriented the responses were. It felt like the students had ‘recommendations’ that could be implemented if they had the opportunity to voice their opinions and concerns.

Exploring the influence that the context may have on the development of creativity, the current study shows some support for findings by Davies et al. (2013) since respondents referred to physical, pedagogical and also the external environment and the subsequent factors referred to by the authors. Respondents appeared to place some emphasis on their preference for calm surroundings where more greenery could be enjoyed perhaps outside the physical classroom. In relation to the context in school, of particular importance are emerging findings relating to the need for students to have access to down-time and quiet spaces and a wish for more autonomy.

Although we are pleased with the outcome of the study, some limitations need to be acknowledged. These limitations were related to the extent of data that was collected and the data collection method. Due to restricted access in schools induced by the COVID-19 pandemic at the time of data collection, online methods were used. It would have been interesting to access the school and follow up with interviews of focus groups. Moreover, psychological factors including openness to experience, conscientiousness and agreeableness were not included in this study. It would have been interesting to see how these constructs could relate to CPI in a sample population so young. Moreover, it would be of interest to see what the results of a longitudinal study would be as the young students grow into adolescents and young adults. Exploring the role of the creators and how it evolves in changing contexts could inform how creativity can best be harnessed to enhance the capabilities of young people in an effort to further develop their 21st Century Skills.

## 6. Conclusions

Findings from the two studies indicate that young students have adequate interpretations of the term ‘creativity’ while statistical tests revealed that the sample population scored highly on their self-perceived CPI. Schools are often referred to as places where creativity fades, however, plenty of opportunities exist to enhance engagement with skills that promote it in its different aspects.

## Figures and Tables

**Table 1:** Items used from the Short Scale of Creative Self (adapted from Karwowski et al. 2018).

How Well Do the Following Statements Describe You?						
I think I am a creative person	No way	Not really	Maybe	A little bit less	Yes	No response
My creativity is important for who I am	No way	Not really	Maybe	A little bit less	Yes	No response
Being a creative person is important to me	No way	Not really	Maybe	A little bit less	Yes	No response
Creativity is an important part of myself	No way	Not really	Maybe	A little bit less	Yes	No response
Finding a solution is a characteristic which is important to me	No way	Not really	Maybe	A little bit less	Yes	No response

**Table 2:** Open and Close-ended questions used.

Open-Ended Questionnaire:
What is creativity?
Are you creative?
When are you creative most?
Is school a place where you can be creative?
When are you most creative during the school day?
What would help you to be more creative while at school?
What was the most creative thing you ever did at school?
Why do you think it was creative?

**Table 3:** Six-Phases Approach by Braun and Clarke (2006).

Thematic Analysis
Phase 1: familiarizing yourself with the data
Phase 2: generating initial codes
Phase 3: searching for themes
Phase 4: reviewing themes
Phase 5: defining and naming themes
Phase 6: producing the report

**Table 4:** Examples for descriptions developed for each generated code.

Code	Description
Creativity = Imagination	Creativity defined by the participants as imagination, including dreaming and inventing.
Creativity = Fun	Creativity described as being fun, cool, awesome, enjoyable, relaxing. Also to “make something boring a bit more fun”.
Creativity = Self-Expression	Creativity as a form of self-expression (e.g., it makes me feel happy, doing something I’m excited for, it is important to me) and judging the outcome as being creative (e.g., it came really pretty, it was colorful etc.). Also growth (e.g., “helped to believe in myself more”).

**Table 5:** Creative Personal Identity.

CPI Item	*n*	Mean	SD
I think I am a creative person	557	4.30	0.990
My creativity is important for who I am	551	4.40	0.991
Being a creative person is important to me	555	4.47	0.946
Creativity is an important part of myself	556	4.42	0.979
Finding a solution is a characteristic which is important to me	557	4.26	0.975
CPI Average	538	4.37	0.685

**Table 6:** Regressions (State Schools and Church Schools; dependent variable = CPI average).

	B	S.E.	t
Gender	.000	.063	.003
Class	−.159 **	.065	−2.438
Number of Siblings	−.052	.038	−1.354
State School	.223 *	.090	2.474
Church School	.115	.070	1.642
Constant	4.564 ***	.159	28.670
*R Square*	.028 *		
*F*	2.691 *		

*** *p* < 0.001; ** *p* < 0.01; * *p* < 0.05 (2-tailed).

**Table 7:** Summary of emerging findings—Overarching Theme Creator.

Sub-Themes	Categories	Codes
Implicit definitions of creativity	Mental processes	Imagination, dreaming, inventing, having fun
	Thinking and doing	Creating something, working on a project, creating workarounds, working with limitations
Students’ perceptions of creativity in relation to the academic definitions of the term	Novelty	Innovativeness, newness
	Originality	Uniqueness, outstandingness
	Usefulness	Relevance to others, importance
Individual characteristics that make up creators and their self-perception	Personality	Character, talent, form of self-expression, specific needs (e.g., being alone or with others, need for autonomy)
	Skill	Abilities, knowledge
Attributes of creations by the creators	Qualities	Tangible, functional, look (e.g., pretty, colorful)
Elements that may catalyze/nurture creativity	Conditions	Input, support, autonomy, freedom, silence, time to think and concentrate, time to play

**Table 8:** Summary of emerging findings—Overarching Theme Context.

Sub-Themes	Categories	Codes
Different places where the students are creative	At school	Subject (e.g., science, P.E., etc.), creative activities (e.g., writing, drama, etc.)
	Outside of the school environment	At home, leisure time, idle time, extra-curricular activities, breaks
Stimulation through the environment that fosters the student’s creativity	At school	Materials and supplies (e.g., craft items, paper, colors, etc.)
	Outside of the school environment	Nature, plants, music

## Data Availability

The data that support the findings of this study are available from the corresponding author upon reasonable request.

## Supplementary Materials

The following supporting information can be downloaded at: https://www.mdpi.com/article/10.3390/jintelligence11030053/s1, Table S1 Correlation Analysis, Table S2 Questionnaire.
