# Supplementary material for: Young Creators: Perceptions of Creativity by Primary School Students in Malta
Source: J Intell. 2023 Mar 13;11(3):53. doi: 10.3390/jintelligence11030053 (PMC10053983; doi:10.3390/jintelligence11030053)
Supplement: Supplementary file 1 [file jintelligence-11-00053-s001.zip › jintelligence-2213881-supplementary.pdf]

Journal of Intelligence

Supplementary Material

Young Creators: Perceptions of Creativity by Primary School Students in Malta

Table S1. Correlation Analysis

	1	2	3	4	5	6	7	8	9	10	11	12	13	14	15	16	17	18	19	20	21
**1**	1																				
**2**	-0.3900*	1																			
**3**	-0.3333*	-0.7382*	1																		
**4**	-0.0663	-0.0008	0.0494	1																	
**5**	0.0663	0.0008	-0.0494	-1	1																
**6**	-0.0396	0.082	-0.0549	-0.007	0.007	1															
**7**	0.0217	-0.0568	0.0423	0.2124*	-0.2124*	-0.1186	1														
**8**	0.1075	0.0028	-0.0816	-0.064	0.064	0.0589	-0.2366*	1													
**9**	-0.0611	0.0731	-0.0301	0.0682	-0.0682	0.0474	-0.2447*	-0.0946	1												
**10**	0.0954	0.0148	-0.085	-0.0862	0.0862	0.0271	-0.0824	-0.0279	0.0013	1											
**11**	-0.1255	0.1156	-0.0264	-0.0321	0.0321	-0.0617	0.0706	-0.1094	-0.0263	-0.2197*	1										
**12**	0.0892	-0.1225	0.0601	0.0664	-0.0664	0.0516	-0.0389	0.1214	0.0261	-0.1715*	-0.9234*	1									
**13**	-0.0696	-0.1528	0.2074*	0.0242	-0.0242	-0.0537	-0.0105	0.035	0.0756	0.0312	-0.119	0.1079	1								
**14**	0.1175	-0.1323	0.0494	0.2069*	-0.2069*	0.0672	0.0543	0.0046	0.005	0.0125	-0.1227	0.119	-0.6186*	1							
**15**	0.0862	-0.0349	-0.0275	-0.0384	0.0384	-0.0216	-0.0083	0.0885	-0.0516	0.0275	-0.0286	0.0181	-0.1129	-0.3425*	1						
**16**	0.0252	-0.0375	0.0198	0.0356	-0.0356	0.0187	-0.0354	-0.0196	0.0324	0.0601	0.0058	-0.0295	-0.0256	0.0668	-0.0295	1					
**17**	0.0475	0.0321	-0.0677	0.1024	-0.1024	-0.0688	-0.0243	-0.0056	0.0698	0.0391	0.0481	-0.0639	-0.0733	0.1102	-0.1408	0.3699*	1				
**18**	0.0574	0.0898	-0.1338	0.0721	-0.0721	-0.076	-0.0094	0.0073	0.0479	0.0267	0.0571	-0.0681	0.0135	0.0107	-0.108	0.3507*	0.5080*	1			
**19**	0.018	0.0746	-0.0894	0.1249	-0.1249	-0.0837	-0.0039	-0.0189	0.0797	0.0255	0.0687	-0.0794	-0.0056	-0.0226	-0.0229	0.4479*	0.5528*	0.6398*	1		
**20**	0.0661	0.0127	-0.0615	0.0042	-0.0042	-0.0265	0.0126	-0.0292	0.046	0.0492	-0.0265	0.0074	-0.0249	0.0075	0.0123	0.1367	0.2089*	0.2497*	0.2769*	1	
**21**	0.0561	0.0595	-0.1017	0.1058	-0.1058	-0.0783	-0.006	-0.0174	0.0772	0.0532	0.0558	-0.0775	-0.0336	0.0501	-0.0984	0.6364*	0.7760*	0.7949*	0.8530*	0.4534*	1

1 = State Schools; 2 = Church Schools; 3 = Independent Schools; 4 = School Year 5; 5 = School Year 6; 6 = Sector OQ; 7 = Favorite Subject Mathematics; 8 = Favorite Subject English; 9 = Favorite Subject Sciences; 10 = Online Delivery of Classes; 11 = Classes at School; 12 = Hybrid Delivery of Classes; 13 = No Siblings; 14 = 1-2 Siblings; 15 = 3 or more Siblings; 16 = Item 1 CPI; 17 = Item 2 CPI; 18 = Item 3 CPI; 19 = Item 4 CPI; 20 = Item 5 CPI; 21 = Overall CPI Score

Table S2. Questionnaire

Section 1: Demographic Data<br>Which school are you at?<br>State<br>Church<br>Independent<br>Which year are you in?<br>Year 5<br>Year 6<br>What job do the people who look after you do?<br>What is your favorite subject?<br>Which one best describes your experience at school?<br>All lessons online<br>All lessons are at school<br>We have a mix of online and face-to-face lessons<br>How many brothers and sisters do you have?
Section 2: SSCS Items for Creative Personal Identity (CPI)<br>I think I am a creative person<br>My creativity is important for who I am<br>Being a creative person is important to me<br>Creativity is an important part of myself<br>Finding a solution is a characteristic which is important to me
Section 3: Open- and closed-ended questions<br>What is creativity?<br>Are you creative?<br>When are you creative most?<br>Is school a place where you can be creative?<br>When are you most creative during the school day?<br>What would help you to be more creative while at school?<br>What was the most creative thing you ever did at school?<br>Why do you think it was creative?
